# The influence of cations on α-lactalbumin amyloid aggregation

**DOI:** 10.1007/s00775-022-01962-3

**Published:** 2022-09-23

**Authors:** Andrea Antosova, Miroslav Gancar, Zuzana Bednarikova, Jozef Marek, Eva Bystrenova, Zuzana Gazova

**Affiliations:** 1 Institute of Experimental Physics Slovak Academy of Sciences, Watsonova 47, 040 01 Kosice, Slovakia; 2 Consiglio Nazionale Delle Ricerche, Istituto Per Lo Studio Dei Materiali Nanostrutturati (CNR-ISMN), via P. Gobetti 101, 40129 Bologna, Italy

**Keywords:** α-Lactalbumin, Amyloid aggregation, Cations, Kinetics, Morphology

## Abstract

There is limited knowledge regarding α-lactalbumin amyloid aggregation and its mechanism. We examined the formation of α-lactalbumin amyloid fibrils (α-LAF) in the presence of cations (Mg^2+^, Ca^2+^, Na^+^, K^+^, NH_4_^+^, and Cs^+^) in the form of chloride salts at two concentrations. We have shown that studied cations affect the conformation of α-lactalbumin, the kinetics of its amyloid formation, morphology, and secondary structure of α-LAF in a different manner. The higher salts concentration significantly accelerated the aggregation process. Both salt concentrations stabilized α-lactalbumin's secondary structure. However, the presence of divalent cations resulted in shorter fibrils with less β-sheet content. Moreover, strongly hydrated Mg^2+^ significantly altered α-lactalbumin's tertiary structure, followed by Na^+^, NH_4_^+^, K^+^, and weakly hydrated Cs^+^. On the other hand, Ca^2+^, despite being also strongly hydrated, stabilized the tertiary structure, supposedly due to its high affinity towards α-lactalbumin. Yet, Ca^2+^ was not able to inhibit α-lactalbumin amyloid aggregation.

**Graphic abstract:**

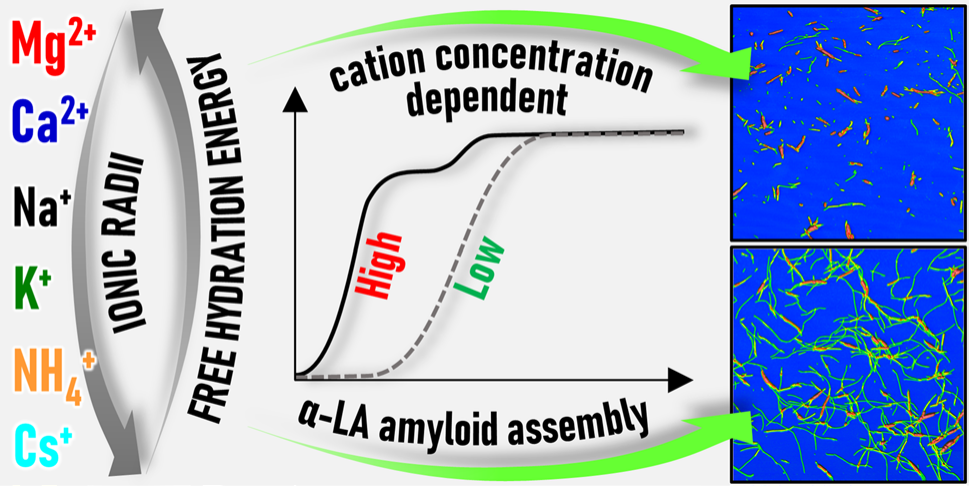

**Supplementary Information:**

The online version contains supplementary material available at 10.1007/s00775-022-01962-3.

## Introduction

α-Lactalbumin (α-LA) is a small (123 amino acids), acidic (pI 4–5), globular protein found in the whey fraction of milk in all mammals [1] as a component of lactose synthetase [2]. The α-LA molecule consists of a large α-helical domain and a small, mostly β-structured domain connected by a calcium-binding loop. This single Ca^2+^-binding site can also bind Mg^2+^, Mn^2+^, Na^+^, K^+^, and other metal cations. Moreover, it possesses several distinct Zn^2+^ -binding sites. The physiological role of α-LA's interaction with Ca^2+^ and other metal cations is still unknown. However, α-LA's physical properties strongly depend on the occupation of its metal-binding sites by metal ions, since in their absence α-LA acquires the molten globule-like state [3]. The binding of metal ions, especially Ca^2+^, increases the stability of α-LA against heat, various denaturing agents, and proteases and influences refolding and disulfide bond formation in the denatured α-LA [4]. α-LA and some of its fragments possess bactericidal and antiviral activities [5]. Complexes of partially unfolded α-LA with oleic acid showed significant cytotoxicity to various tumors and bacteria, with α-LA playing a role of a delivery carrier of cytotoxic fatty acid molecules across the cell membrane [6].

From the biotechnology point of view, α-LA can form nanoparticles, nanotubes, and fibers depending on experimental conditions. Protein-based nanoparticles can be applied in the pharmaceutical and food industries. For example, spheroidal nanoparticles (100–200 nm in diameter) from bovine α-LA cross-linked with glutaraldehyde in the presence of acetone were prepared [7]. The formation of nanotubes from partially hydrolyzed α-LA was investigated at various pH values, concentrations, and calcium levels. Short α-LA nanotubes exhibited excellent permeability in mucus, enabling them to quickly reach epithelial cells and deliver active compounds [8]. In food science, in vitro fibrillization of so-called food proteins was suggested as an approach to produce new ingredients for food formulations. These amyloid fibrils contain diverse reactive functional groups on their surface, making them tunable by numerous agents. It was published that food protein fibrils may be used as the next generation of biomimetic cell culture platforms [9] and have great potential as nano-biosorbents for the rapid and efficient removal of pollutants [10].

The design of amyloid-based templates with desired properties requires detailed knowledge about amyloid self-assembly mechanisms. Amyloid aggregation of α-LA has not been studied as extensively compared to some other proteins. Therefore, many questions still need to be answered to understand its mechanism. In general, amyloid aggregation of various proteins can be readily modulated by the presence of salts. To date, the majority of investigations on α-LA amyloid fibrillization have only used NaCl. It has been found that Ca^2+^-depleted α-LA forms amyloid fibrils in vitro from its molten globular state induced by low pH 2.0 and the presence of 100 mM NaCl [11]. Kurouski et al*.* prepared amyloid fibrils using Ca^2+^-depleted α-LA, incubating the protein at different pH values in the presence of 150 mM NaCl at 37 °C for 3 days [12]. It has been shown that ions might modulate the processes of amyloid aggregation through nonspecific Debye–Hückel screening, site-specific salt bridging by polyvalent ions, and/or competing with protein for preferential interactions with water, so-called "specific ion effects" [13]. Studying the effects of the cations is specifically relevant since they occur ubiquitously in the physiological environment and play an essential role in amyloid fibril formation [14, 15]. It was further reported that various ions (Mg^2+^ and SO_4_^2−^ in particular) promote the amyloid formation of several proteins such as lysozyme, immunoglobulin light chain, B1 domain of protein G, β_2_-microglobulin, α-synuclein, and Aβ_40_ [14, 16–19].

In this work, we have sought to better understand various cations’ effects on fibril network assembly, with possible utilization as future bionanomaterials.

## Materials and methods

α-Lactalbumin from bovine milk (lyophilized powder, type III, calcium depleted, purity ≥ 85%, lot number L6010), NaCl, KCl, CsCl, NH_4_Cl, CaCl_2_, MgCl_2_, HCl, thioflavin T (ThT), were obtained from Sigma Aldrich Chemical Company (St. Louis, MO). All other chemicals were obtained from Sigma Aldrich Chemical Company (St. Louis, MO) and were of analytical grade. Ultra-pure deionized water (Milli-Q) was used throughout the experiments.

### Formation of α-lactalbumin amyloid fibrils (α-LAF)

α-LA was dissolved to a final concentration of 5 mg ml^−1^ in 100 mM and 300 mM salts (MgCl_2_, CaCl_2_, NaCl, KCl, NH_4_Cl, CsCl). Protein solutions' pH was adjusted to 2.0 by the addition of HCl. The formation of amyloid fibrils was achieved by incubating the solution at 65 °C and constant stirring (1200 rpm) for 75 min. The formation of amyloid fibrils was confirmed using ThT assay and atomic force microscopy (AFM).

### Thioflavin T (ThT) fluorescence assay

The formation of α-LAF was monitored by characteristic changes in ThT fluorescence intensity. ThT was added to studied α-LAF samples (10 µM) to a final concentration of 20 μM, and samples were incubated at 37 °C for 1 h. Fluorescence intensity was measured in a black 96-well plate using a Synergy Mx (BioTek Company, USA) spectrofluorometer. The excitation wavelength was set at 440 nm, and the emission was recorded at 485 nm. The emission/excitation slits and offset were adjusted to 9.0/9.0 nm and 6 mm, respectively. All ThT fluorescence experiments were performed as triplicate, and the final value represents the average of measured values with related deviations.

### Morphology of amyloid aggregates—atomic force microscopy (AFM) and image analysis

AFM samples (3 μM protein concentration) were prepared by drop-casting of 10 μl sample aliquots on a freshly cleaved mica surface followed by a 5 min adsorption period. Samples were afterward rinsed five times using ultrapure water and nitrogen-dried. The images were taken using an atomic force microscope NT-MDT Smena in a tapping mode under ambient conditions using rectangular antimony doped, single crystal silicon (*n*-type) probe NSG01 (gold-coated reflective side) with a resonance frequency of 87–230 kHz and a force constant of 1.45–15.1 N m^−1^. AFM topographic images were corrected line-by-line for background trend effect with second-order polynomial fitting using Gwyddion software (v. 2.55) [20].

### Kinetics of HEWL amyloid fibrillization

The kinetics of α-LAF fibril formation was studied by ThT measurements. The amyloid fibrillization was induced by conditions described in paragraph *Formation of α-lactalbumin amyloid fibrils (α-LAF)*. Aliquots (3 μl) were withdrawn from samples at given times. Each experiment was performed as a quintuplicate, and the reported values represent the average of measured data. Corresponding error bars stand for the average deviation.

### Analysis of α-LA amyloid fibrillization kinetics data

The kinetics of α-LA aggregation was represented by changes in the ThT fluorescence intensity plotted as a function of time. Experimental data for α-LA aggregation in the presence of 100 mM salts were subsequently analyzed by nonlinear regression using a standard sigmoidal described by Eq. (1), and data collected for α-LA aggregation in the presence of 300 mM salts were fitted by a two-state sigmoidal function described by Eq. (2):

1 $$y={f}_{\mathrm{Base}}+\frac{{f}_{\mathrm{Max}}-{f}_{\mathrm{Base}}}{1+\mathrm{exp}\left(-{k}_{\mathrm{Agg}}\left(t-{t}_{\mathrm{Half}}\right)\right)}$$

2 $$f\left(t\right)={f}_{\mathrm{Base}}+{f}_{1}\left(t\right)+{f}_{2}\left(t\right)$$

$${f}_{1}\left(t\right)=\frac{{f}_{\mathrm{Mid}}-{f}_{\mathrm{Base}}}{1+\mathrm{exp}\left(-{k}_{{\mathrm{Agg}}^{1}}\left(t-{t}_{{\mathrm{Half}}^{1}}\right)\right)}$$

$${f}_{2}\left(t\right)=\frac{{f}_{\mathrm{Max}}-{f}_{\mathrm{Mid}}}{1+\mathrm{exp}\left(-{k}_{{\mathrm{Agg}}^{2}}\left(t-{t}_{{\mathrm{Half}}^{2}}\right)\right)}$$

$${k}_{{\mathrm{Agg}}^{1}} = 2 / ({t}_{{\mathrm{Half}}^{1}} - {t}_{{\mathrm{Lag}}^{1}})$$

$${k}_{{\mathrm{Agg}}^{2}} = 2 / ({t}_{{\mathrm{Half}}^{2}} - {t}_{{\mathrm{Lag}}^{2}})$$

where *f*_Base_ is the baseline fluorescence intensity, *f*_Mid_ is the first fluorescence intensity saturation, *f*_Max_ is the final fluorescence intensity saturation, *t* is the time, *t*_Half_^1^, *t*_Half_^2^ represents the time at half-height of the first and second transition, *k*_Agg_^1^, *k*_Agg_^2^ are aggregation rate constants and *t*_Lag_^1^, *t*_Lag_^2^ are corresponding lag times [21, 22]. The accuracy of the fit was monitored by the correlation parameter *R*^2^ ≥ 0.992 in all cases.

### Determination of the secondary structure of α-LA and its aggregates by ATR-FTIR spectroscopy

ATR-FTIR spectra of α-LAF samples at a concentration of 5 mg ml^−1^ were recorded with a Nicolet 8700 FTIR spectrometer (Thermo Scientific) equipped with a Smart OMNIC sampler (diamond prism). Each spectrum represents an average of 254 interferograms recorded in the amide I region (1700–1600 cm^−1^) with a resolution of 2 cm^−1^. Each sample was measured as a triplicate (*n* = 3), and the representative spectra were selected for the manuscript. The buffer background was subtracted from the spectrum. Origin 8.5 Pro was used to deconvolve the recorded ATR-FTIR spectra and determine the samples’ secondary structure. The precision of the fit was monitored by adjusted *R*^2^ parameter ≥ 0.995 in all cases.

### Monitoring tertiary structure changes of α-LA by near-UV CD spectroscopy

CD measurements of 1 mg ml^−1^ α-LA samples were performed using a JASCO-815 spectropolarimeter. The reported CD spectra represent the average of five cumulative scans, and every sample has been measured as a triplicate (*n* = 3). The measurements in the near-UV region (250–320 nm) with a 0.2 nm step size were performed in a rectangular quartz cuvette (1 cm optical path) with a scan rate of 50 nm min^−1^ at 25 °C. Weighted spectral difference analysis (WSD) was performed following the protocol by Dinh et al*.* [23]. In short, this method yields a single WSD value as a measure of similarity between two spectra according to the following Eq. (3):

3 $$\mathrm{WSD}= \sqrt{\sum_{i=1}^{n}\left[\left(\frac{1}{n}\right)\left(\frac{\left|{y}_{Ai}\right|}{{\left|{y}_{A}\right|}_{ave.}}\right){\left({y}_{Ai}-{y}_{Bi}\right)}^{2}\right]}$$

where *y*_*Ai*_ is the reference spectrum, and *y*_*Bi*_ represents a sample spectrum. First, the sample spectrum is subtracted from the reference spectrum, and the resulting difference spectrum is squared. A weighting based on the reference is then applied to this squared difference. Absolute reference values are normalized by dividing through their average and multiplied by the squared difference spectrum, obtaining a weighted difference spectrum. The final WSD value is obtained by calculating the average intensity of the weighted difference spectrum by summing over all data points (*i*), dividing by their total number (*n*), and taking its square root. The presented spectra were smoothed by the Spectral Manager analysis program and Origin 8.5 Pro, respectively.

### Polyacrylamide gel electrophoresis (SDS-PAGE)

Samples from the first plateau phase of α-LA amyloid fibril formation were evaluated by SDS-PAGE (12% acrylamide wt/vol). Samples collected from ThT kinetics (13–20 min) were mixed with loading buffer (100 mM Tris–HCl, pH 6.8, 4% w/v SDS, 0.2% v/v bromophenol blue, 20% v/v glycerol) to a final protein concentration of 10 μg per well. Samples were loaded in the gel, and the experiment ran for ~ 3.5 h at a constant current of 50 mA, max. voltage of 150 V, and max. power of 10 W. After staining (0.25% Coomassie brilliant blue (G-250) in 50% v/v methanol and 10% v/v glacial acetic acid), the gels were destained in the solution consisting of methanol: glacial acetic acid: water in the ratio of 50:40:10, respectively.

## Results

In the present work, we have studied the formation of α-LA amyloid fibrils (α-LAF) in the presence of various chloride salts, namely MgCl_2_, CaCl_2_, NaCl, KCl, NH_4_Cl, and CsCl. We focused on the effect of cations due to their different physicochemical properties, namely ionic radii in water (*R*) and free hydration energy (*− Δg*_hydr_) (Table S1) [24, 25]. Furthermore, we have compared the effect of two salt concentrations: 100 mM, which is widely used in the in vitro preparation of amyloid aggregates, and 300 mM, at which the observed impact of ions ought to be more pronounced.

### Kinetics of α-LA amyloid formation

**Fig. 1:**
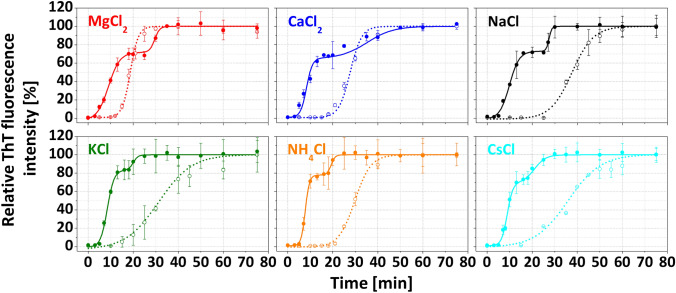
Effect of different salts on amyloid aggregation kinetics of α-LA: MgCl_2_ (red), CaCl_2_ (*blue*), NaCl (*black*), KCl (*green*), NH_4_Cl (*orange*) and CsCl (*cyan*) at 100 mM (*dotted lines*, empty symbols) and 300 mM (*solid lines*, full symbols) concentrations. *Error bars* represent the average deviation of three separate measurements

The effect of 100 mM and 300 mM studied chloride salts on the kinetics of α-LA fibrillization has been examined using a thioflavin T (ThT) fluorescence assay (Fig. 1). ThT is an amyloid-specific dye whose fluorescence increases upon binding to cross-β motif characteristic for amyloid fibrils [26]. α-LA does not form amyloid fibrils within 24 h in the absence of salts (results not shown). The addition of salts promoted fibrillization significantly as all samples showed increased ThT fluorescence following a sigmoidal/double sigmoidal growth curve containing well-resolved lag, growth, and plateau phases (Fig. 1). By analyzing given curves, kinetic parameters of aggregation such as lag time *t*_Lag_ (the nucleation propensity of a sample), aggregation constant *k*_Agg_ (the elongation rate of amyloid fibrils), and *t*_Half_ (the half-time of aggregation) were derived (Table 1 and S2).

**Table 1:** Kinetic parameters derived from aggregation kinetics of α-LA in the presence of 100 mM salts

100 mM salts	*t*_Lag_ (min)	*t*_Half_ (min)	*k*_Agg_ (min^−1^)
MgCl_2_	14.2 ± 0.4	18.5 ± 0.2	0.47 ± 0.04
CaCl_2_	19.9 ± 0.6	27.5 ± 0.3	0.26 ± 0.02
NaCl	28.0 ± 1.2	37.6 ± 0.6	0.21 ± 0.02
KCl	21.0 ± 2.6	30.5 ± 1.2	0.21 ± 0.02
NH_4_Cl	22.6 ± 0.6	29.8 ± 0.4	0.28 ± 0.03
CsCl	21.3 ± 4.3	35.8 ± 1.7	0.19 ± 0.03

Every experiment was performed as a triplicate, and the final data represent an average value accompanied by a corresponding deviation. *t*_Lag_ represents a lag time of aggregation, *t*_Half_ stands for half-time of aggregation, and *k*_Agg_ is the aggregation rate constant

In the case of 100 mM salts, the fibrillization is described by the sigmoidal growth curve (dotted lines in Fig. 1). The lag phase duration increased in the order MgCl_2_ < CaCl_2_ < KCl < CsCl < NH_4_Cl < NaCl. The kinetics of α-LAF formation in the presence of divalent salt MgCl_2_ was faster compared to the CaCl_2_ and monovalent salts, as evidenced by the shortest *t*_Lag_ (14.2 ± 0.4 min) and the highest *k*_Agg_ (0.47 ± 0.04 min^−1^). These results are in good agreement with the previous studies showing that MgCl_2_ significantly accelerated the formation of Aβ_40_ and β-lactoglobulin fibrils [14, 27]. We determined comparable kinetic parameters for cations K^+^, NH_4_^+^, Cs^+^, and Ca^2+^, particularly lag times. The slowest aggregation was observed in the presence of NaCl (*t*_Lag_ = 28.0 ± 1.2 min, *k*_Agg_ = 0.21 ± 0.02 min^−1^). Such behavior may be related to the fact that Na^+^ has a relatively low − *Δg*_hydr_ and small radius. Moreover, as Arakawa et al*.* noted, Na^+^ leads to the highest salting-out effect and protein stability, protecting it against denaturation [28].

In general, the salt concentration is a crucial factor influencing amyloid fibrillization, given that salts affect various interactions in the process. In our experiments, increasing salt concentration from 100 to 300 mM accelerated α-LA amyloid fibril formation (Fig. 1, full lines), which could be characterized by double sigmoidal growth curves. All studied cations caused a significant shortening of the time required to form mature fibrils compared to lower salt concentration, as evidenced by kinetic parameters *t*_Lag_^1^ and *t*_Half_^1^ determined for the first sigmoidal curve (Table S2). The *t*_Lag_^1^ increased in the order CaCl_2_ ≤ MgCl_2_ < KCl < NaCl < NH_4_Cl < CsCl. The second sigmoidal curves of the fibrillization were also characterized by kinetic parameters (Table S2). MgCl_2_, CaCl_2_, and NaCl significantly increased *t*_Lag_^2^ and *t*_Half_^2^ compared to other studied salts.

### Morphology of α-LA aggregates

**Fig. 2:**
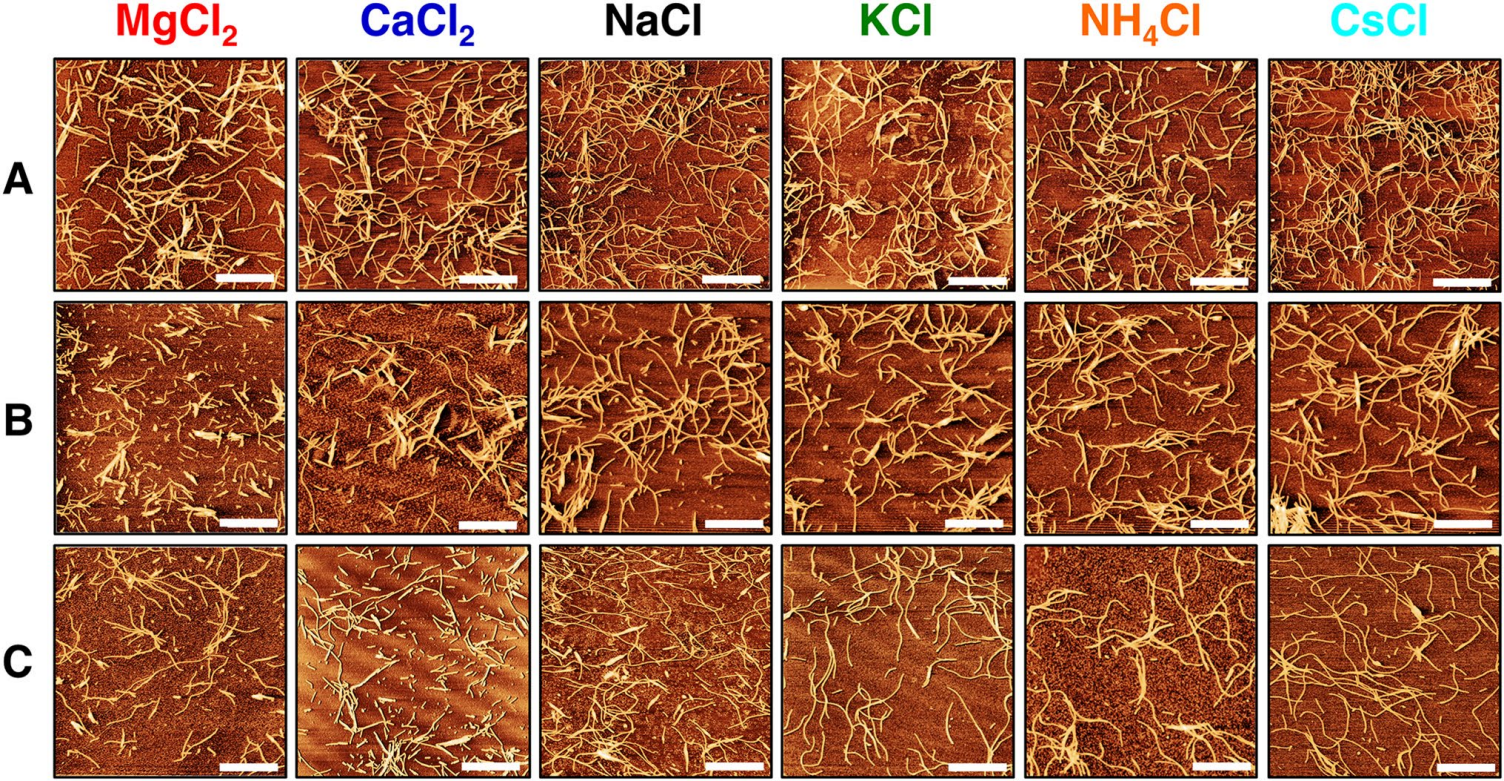
AFM images of α-LA amyloid structures prepared in 100 mM (row **A**), 300 mM salts after 75 min incubation (row **B**) and at times corresponding to the first plateau phase of double sigmoidal growth curves (Table S5) (row **C**). All images are 5 × 5 μm with scale bar representing 1.25 μm. The topographic images were corrected line-by-line for background trend effect with second-order polynomial fitting

α-LA aggregates formed in the presence of studied salts at two concentrations were visualized using AFM. Interestingly, observed differences in kinetics did not affect the overall morphology of amyloid fibrils prepared in 100 mM salts (Fig. 2, row A). AFM images obtained for 300 mM salts concentrations at the plateau phase of the second sigmoidal curve (*t* = 75 min) suggest that the morphology of α-LAF fibrils varied (Fig. 2, row B). The most significant differences were observed for divalent salts. In the presence of 300 mM MgCl_2_, fibrils are shorter, indicating that a higher MgCl_2_ concentration leads to interference with fibril elongation or may cause gradual fibril fragmentation. We have observed shorter fibrils in the case of 300 mM CaCl_2_ (Fig. 2, row B) compared to long and more flexible fibrils prepared in the presence of monovalent cations. We suggest that these observations might result from the fact that α-LA's metal-binding site preferentially and very strongly binds Ca^2+^ followed by Mg^2+^ [4]. The morphology of α-LAF prepared in the presence of 300 mM monovalent studied cations was visually comparable with fibrils formed in 100 mM salts concentration.

### The α-LA and α-LAF secondary structure content analysis in the presence of salts

**Fig. 3:**
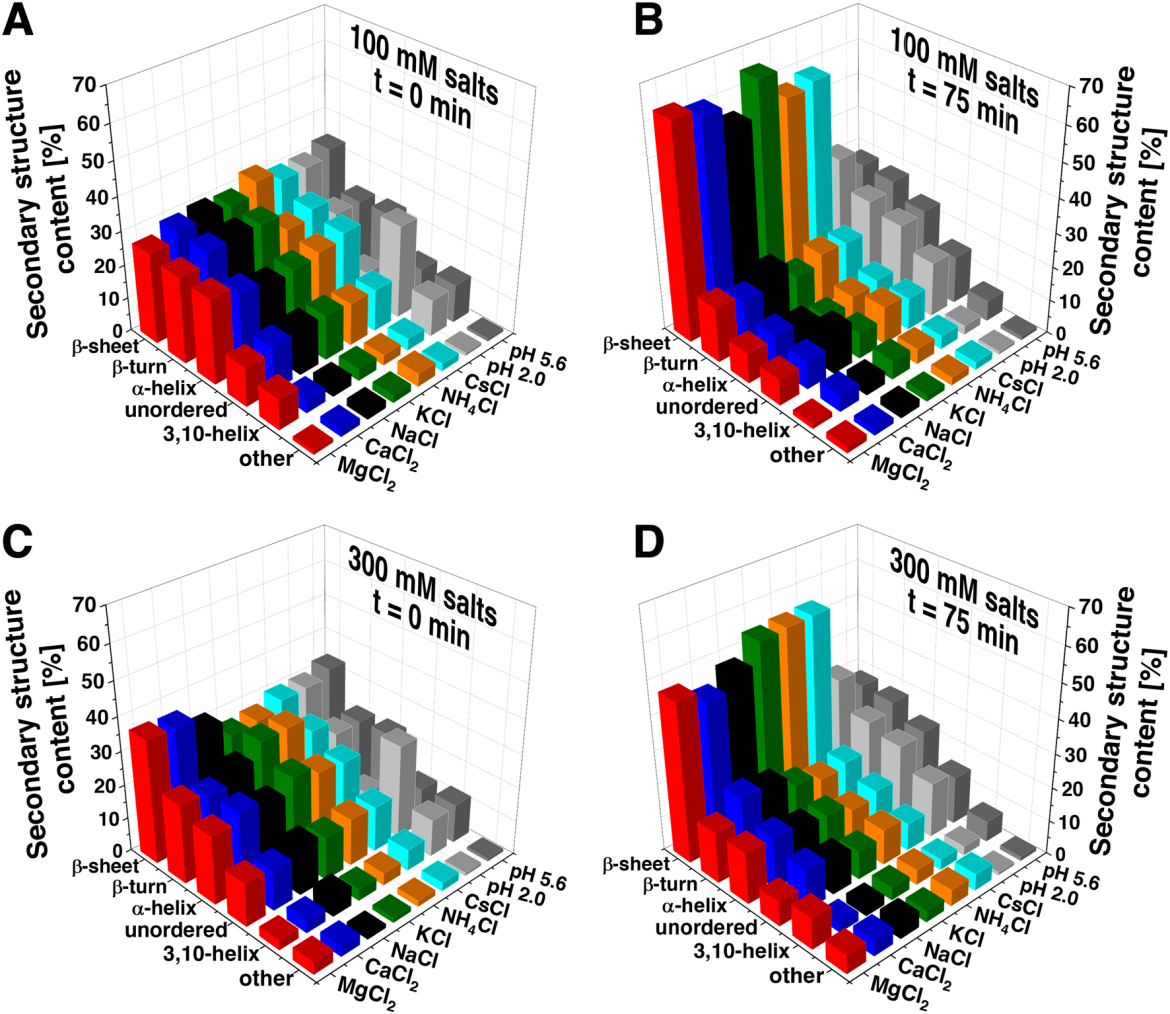
Representation of secondary structure distributions deconvolved from ATR-FTIR spectra of α-LA at *t* = 0 min (**a**, **c**) and α-LAF at *t* = 75 min (**b**, **d**). Samples were prepared in the presence of 100 mM (**a**, **b**) and 300 mM (**c**, **d**) salts: MgCl_2_ (*red bars*), CaCl_2_ (*blue bars*), NaCl (*black walls*), KCl (*green bars*), NH_4_Cl (*orange bars*) and CsCl (*cyan bars*). α-LA dissolved in the water pH 2.0 (*light gray bars*) and pH 5.6 (*dark gray bars*) served as a control for *t* = 0 min (**a**, **c**). α-LA exposed to amyloid inducing conditions in water pH 2.0 (*light gray bars*) and pH 5.6 (*dark gray bars*) served as a control for *t* = 75 min (**b**, **d**)

ATR-FTIR measurements in the amide I region (1600–1700 cm^−1^) were performed to assess the changes in the secondary structure of the α-LA before and after fibrillization in the presence of studied salts (Fig. S2). Measured spectra were subsequently deconvolved, and obtained data are presented in Fig. 3 and Tables S3, S4, and S5. In water (pH 5.6), the secondary structure of native α-LA consists mainly of α-helical and β-sheet/turn content (Fig. 3, dark grey bars and Table S3C and S4C). Decreasing pH (from 5.6 to 2.0) led to the destabilization of α-LA secondary structure as the content of the unordered structure increased (Fig. 3 light grey bars, Table S3C and S4C).

The presence of 100 mM salts at *t* = 0 min and low pH led to an increase in α-helix content (comparable to α-LA in H_2_O, pH 5.6), countering the effect of acidic pH 2.0. Salts stabilized the α-helix mainly at the expense of less stable 3,10-helices, except for Mg^2+^, which also stabilized this type of structure. We have also observed an increase in β-turn content (~ 3–8%) in all cases compared to the control (α-LA in H_2_O, pH 2.0) (Fig. 3a, Table S3A, C).

An impact of 300 mM divalent salts at t = 0 min was more pronounced (Fig. 3c, Table S4A). Mg^2+^ and Ca^2+^ behaved distinctly to the rest of the cations as they seemed to increase the β-sheet content compared to other 300 mM cations, all 100 mM cations and both controls (α-LA in H_2_O, pH 2.0 and 5.6) (Fig. 3c, Table S4A, C).

After fibrillization (*t* = 75 min), ATR-FTIR measurements revealed a high amount of β-sheets at the expense of α-helices, typical for amyloid fibrils formation (Fig. 3b, d, Table S3B). Control samples (α-LA in H_2_O, pH 2.0, and 5.6) after 75 min exposition to amyloid-inducing conditions were also included. No significant changes were observed compared to α-LA in H_2_O, pH 5.6 at *t* = 0 min, confirming the importance of salts in amyloid aggregation (Fig. 3b, d, Table S3D, and S4D).

In the presence of 100 mM salts, the β-sheet content in amyloid fibrils decreased in the order: KCl > MgCl_2_ > CaCl_2_ > CsCl > NH_4_Cl > NaCl (Fig. 3b, Table S3B). It is important to mention that despite the significant difference in the 3,10-helical content at *t* = 0 min in the case of Mg^2+^, all fibrils' final secondary structure content was very similar. Interestingly, fibrils prepared in 100 mM KCl had the highest β-sheet content among the studied salts, however, at the expense of the lowest β-turn and unordered structures (Table S3B).

The β-sheet content after fibrillization in the presence of 300 mM salts decreased in the order: CsCl > NH_4_Cl > KCl > NaCl > MgCl_2_ > CaCl_2_ (Fig. 3d, Table S4B). The amount of α-helices is significantly higher in the case of all studied 300 mM salts compared to α-LAF prepared at 100 mM salts concentration. Therefore, we conclude that 300 mM salts stabilized the α- and 3,10-helices of α-LA more than 100 mM salts, affecting the overall fibrils morphology observed by AFM (Fig. 2, row B). The most significant was the impact of MgCl_2_, as these samples contained ~ 7 – 9% more α-helical structure (mainly 3,10-helix) compared to the other cations (Fig. 3d, Table S4B). Moreover, amyloid fibrils (*t* = 75 min) formed in the presence of 300 mM MgCl_2_ and CaCl_2_ had the lowest β-sheet content (47.2 and 43.7%, respectively) despite having the highest β-sheet content at *t* = 0 min (36.7 and 36.3%, respectively) (Fig. 3d, Table S4B). Another notable observation regarding Mg^2+^ and Ca^2+^ is a steep decrease of β-sheet content in mature fibrils upon increasing the concentration from 100 to 300 mM (from 63.5 and 61.3% to 47.2 and 43.7%, respectively) (Table S3B and S4B).

Based on the findings from infrared spectroscopy measurements, we conclude that a higher concentration of salts/cations influenced the secondary structure of monomeric α-LA (*t* = 0 min), aggregation process, and resulting amyloid α-LAF (*t* = 75 min) to a greater extent, in comparison with 100 mM concentration. Summarized data are presented in Table S5 and Figure S3.

### The double sigmoidal aggregation behavior

To obtain additional morphological/structural insights regarding the observed double sigmoidal aggregation process at 300 mM salt concentration, we have also visualized and performed ATR-FTIR measurements of α-LA amyloid aggregates corresponding to the plateau phase of the first sigmoidal curve. AFM images confirm that amyloid fibrils were formed after ~ 13–20 min incubation in the presence of all studied salts (Fig. 2, row C). α-helical content in the first plateau is lower than in the α-LA at *t* = 0 min and higher than at *t* = 75 min (except for CaCl_2_), pointing to a continuous reorganization of secondary structure towards β-sheets, which is a sign of amyloid fibrillization (Table S5). Based on the collected data, we deduced that in the interval ~ 13–20 min, a high amount of non-aggregated protein is still present in the solution allowing the continuation of the fibrillization process represented by the second sigmoidal curve. To confirm our hypothesis, the composition of samples from the first plateau has been analyzed by SDS-PAGE under partially non-reducing conditions (no added 2-mercaptoethanol). Results show that samples withdrawn in the first plateau phase contain a significant amount of protein weighing ~ 10 kDa (Fig. S1; bands 4–9). Since α-LA weights ~ 14.2 kDa, this observation results from an electrophoretic shift induced by SDS, typical for calcium binding proteins [29], which has also been observed in our control samples: α-LA dissolved in H_2_O pH 2.0 and 5.6 (Fig. S1; bands 2 and 3).

### Tertiary structure of α-LA in the presence of salts

**Fig. 4:**
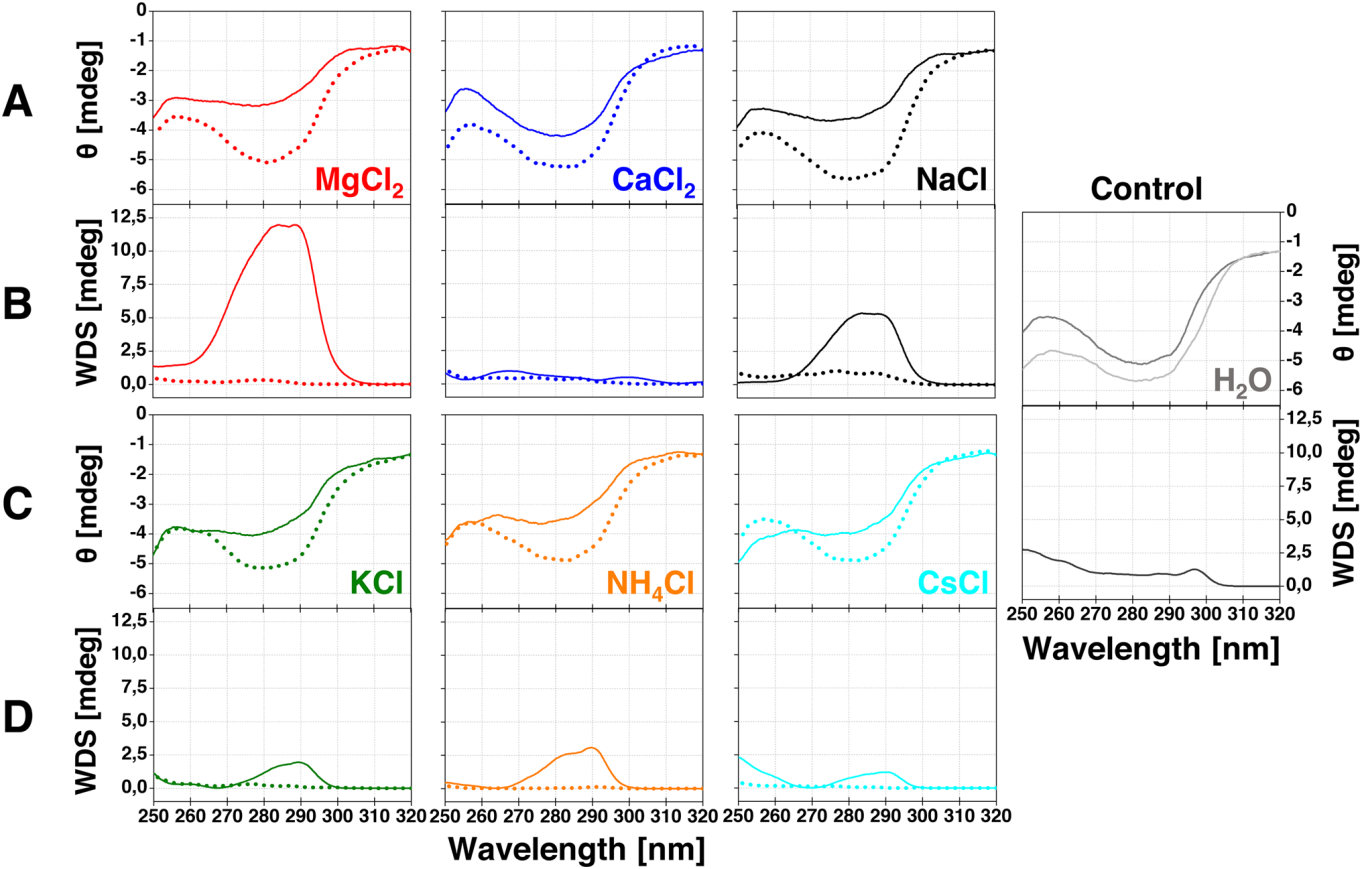
Near UV CD spectra of α-LA (rows **A** and **C**) in the presence of 100 mM (*dotted lines*) or 300 mM (*full lines*) salts: MgCl_2_ (*red lines*), CaCl_2_ (*blue lines*), NaCl (*black lines*), KCl (*green lines*), NH_4_Cl (*orange lines*) and CsCl (*cyan lines*) at pH 2.0, 25 °C, *t* = 0 min. Rows **B** and **D** represent weighted differential spectra (WDS), demonstrating changes in the α-LAs' tertiary structure against α-LA dissolved in the water pH 2.0; 25 °C (light gray line). α-LA dissolved in the water pH 5.6; 25 °C (*dark gray line*) served as a control, while the associated WDS represent a change in the tertiary structure of α-LA induced by the change of the pH

Changes in the immediate environment of aromatic amino acids in proteins can be characterized by near UV-CD measurements (Phe: 255–275 nm, Trp/Tyr: 285–300 nm) [30, 31], making it a sensitive technique to monitor even minor changes in protein tertiary structure. α-LA's structure contains four of each aromatic amino acid. The near-UV CD spectra (Fig. 4, rows A and C) of α-LA in the presence of salts at both studied concentrations were collected at the beginning of the fibrillization process (*t* = 0 min; 25 ℃). Sample spectra were evaluated against the control (α-LA, H_2_O, pH 2.0, 25 °C). CD spectra in the presence of 300 mM salts (Fig. 4, rows A and C, full lines) signify a loss of α-LA's tertiary structure—less pronounced minima and maxima, except for CaCl_2_, contrary to spectra of samples containing 100 mM salts (Fig. 4, rows A and C, dotted lines).

To precisely evaluate and quantify these changes, the obtained spectra were analyzed using the WSD method [23]. Each sample spectrum has been subtracted from the reference spectrum (α-LA, H_2_O, pH 2.0, 25 °C) and squared afterward.

Weighted differential spectra, representing changes in α-LA tertiary structure induced by salts in a particular region (Fig. 4, rows B and D), were acquired by multiplying squared differential spectra by normalized reference spectra values. To further quantify the abovementioned changes, WSD values were derived and summarized in Table 2. Similarly to ATR-FTIR, we have observed a change in the α-LA structure upon transition to acidic pH (Fig. 4, control), represented by the WSD = 0.99. An addition of 100 mM salts did not induce substantial changes in the spectra (Fig. 4, dotted lines) in comparison to the reference sample (H_2_O, pH 2.0, 25 °C), as evidenced by WSD values in the following range: 0.19–0.71. Therefore, the tertiary structure was mostly unaffected by 100 mM salts. However, significant differences were observed for 300 mM salts (Fig. 4, full lines) when compared to the reference sample. α-LA's tertiary structure changed considerably in the case of strongly hydrated Mg^2+^ (WSD = 2.13), followed by Na^+^, NH_4_^+^, K^+^ (WSD = 1.33, 0.91 and 0.73, resp.), and weakly hydrated Cs^+^ (WSD = 0.72). Only negligible changes were detected for α-LA in the presence of Ca^2+^ (WSD = 0.68). We propose that the strong binding of Ca^2+^ resulted in the relative preservation of a compact tertiary conformation of α-LA.

**Table 2:** WSD values quantifying an average change in α-LA tertiary structure in the presence of studied salts

Salts	Weighted spectral difference (WSD) (mdeg)	
	100 mM	300 mM
MgCl_2_	0.37	2.13
CaCl_2_	0.52	0.68
NaCl	0.71	1.33
KCl	0.43	0.73
NH_4_Cl	0.19	0.91
CsCl	0.29	0.72
Control	0.99	

A higher value denotes a more significant change. In the case of the control sample, the WSD value represents a change in the tertiary structure of α-LA induced solely by the pH decrease from 5.6 to 2.0

### Discussion

The information regarding the effect of ions, cations in particular, on the mechanism of amyloid fibril formation is currently eluding and, in many cases, contradictory. The Goers procedure (100 mM NaCl, pH 2.0) is the most common way of α-LA amyloid fibrils preparation [11]. Our goal was to study α-LA amyloid aggregation accompanied by various cations (Mg^2+^, Ca^2+^, Na^+^, K^+^, NH_4_^+^, and Cs^+^) in the form of chloride salts at pH 2.0. We have monitored differences in fibrillization kinetics, final fibril morphology, and secondary and tertiary structure of the α-LA monomer and amyloid fibrils. We have shown that the presence of salts promoted α-LA amyloid fibril formation, agreeing with several other studies dealing with different proteins [14–17]. For example, β_2_-microglobulin forms amyloid fibrils or amorphous aggregates depending on the NaCl concentration at pH 2.5 [32]. Roeters et al*.* have established that the spatial structure of amyloid fibrils related to Parkinson's disease highly depends on salt concentration [33].

Typical sigmoidal curves of α-LA fibrillization with a defined lag, elongation, and plateau phases were observed at 100 mM salts concentration. Kinetic parameters determined that the aggregation has been accelerated in correlation with cations' physicochemical properties.

The studies on β_2_-microglobulin [17] and glucagon [34] report that amyloid aggregation depends on the changes in the water structure caused by salts, agreeing with our data on α-LA amyloid assembly, as strongly hydrated small cations (Mg^2+^, Ca^2+^) proved to be more potent catalysts than weakly hydrated large cations (NH_4_^+^, Cs^+^) (Table S1).

Moreover, we have demonstrated that salts concentration significantly affects fibrillization kinetics, which became more complex at higher cation concentrations. The presence of cations at 300 mM salt concentration significantly accelerated the formation of the fibrils, with growth curves best described by double-sigmoidal profiles. Mukhopadhyay et al*.* observed similar double sigmoidal behavior for Aβ_42_ aggregation and explained it as the attachment of monomers to the fibrils' surface, leading to the formation of new fibrils by branching [35]. Double sigmoidal aggregation kinetics were also detailly examined by Grudzielanek [36], Smirnovas [37], Fodera [38], and Ziaunys [39]. It was shown that the first fluorescence intensity increase in the doubles-sigmoidal curve is likely related to the formation of oligomeric intermediate species capable of binding ThT and this event was reproducible only under certain environmental conditions. However, we were able to consistently reproduce this behavior despite using varying salts. Furthermore, we have visualized amyloid fibrils at both plateaus using AFM (Fig. 2, rows B and C). ATR-FTIR revealed that samples from the first plateau phase contain more α-helical and less β-sheet content than samples from the second plateau (Table S5). SDS-PAGE confirmed the significant amount of non-fibrillar protein (~ 10 kDa due to SDS-induced electrophoretic shift) present in samples from the first plateau (Fig. S1), yet no other intermediates (i.e., oligomers) were observed. An interesting remark elaborating on the observed phenomenon has been noted after analyzing derived kinetic parameters. The effect of cations in the second sigmoidal curve (*t*_Lag_^2^ and *t*_Half_^2^) has been inverted relative to the first sigmoidal curve (*t*_Lag_^1^ and *t*_Half_^1^). Particularly in the presence of Mg^2+^, Ca^2+^, and Na^+^, *t*_Lag_^2^ and *t*_Half_^2^ are significantly longer compared to remaining cations, suggesting hindrance of the nucleation/elongation mechanism in the second part of the aggregation process (Table S2). Thus, based on our results, we propose that amyloid aggregates rich in β-sheet content and the morphology of mature amyloid fibrils were promptly formed until the plateau phase of the first sigmoidal curve. Afterward, we have observed further fibrils formation denoted by the second sigmoidal curve followed by a final plateau.

Similar to results on β-lactoglobulin fibrils published by Loveday et al*.*, the effect of 100 mM salts on the morphology and structure of α-LAFs was insignificant [40]. On the other hand, apart from generally faster kinetics in the presence of 300 mM salts, the impact of divalent cations Mg^2+^ and Ca^2+^ was particularly interesting in terms of aggregates' morphology (short fibrils) and secondary structure (lower β-sheet content), which can be partially explained by the physicochemical properties of studied cations in water (Table S1). It was found that MgCl_2_ has a peculiar effect on protein stability [41] and viscosity [40]. Groups of *Tanford* and *Hippel* have shown that MgCl_2_ and MgSO_4_ act through their affinity to hydrophobic side chains of proteins, interact with the protein's polar surface, and increase protein solubility at high concentrations [42–44]. The lowest β-sheet content and lack of length in the case of fibrils prepared in 300 mM MgCl_2_ and CaCl_2_ may result from these cations' strong hydration and small size. Contrarily, in the presence of 300 mM weakly hydrated, large-sized cations K^+^, Cs^+^ and NH_4_^+^, fibrils with a high amount of β-sheets were formed. Secondary structure analysis showed that for relatively small but weakly hydrated Na^+^, the β-sheets content values represent a borderline between the other cations, agreeing with the previously published data for fibrils prepared in 100 mM NaCl [11].

To understand and explain these findings, near UV-CD spectroscopy was employed to obtain information about α-LA tertiary structure changes. The impact of all 100 mM salts, represented by the derived WSD parameters, was lesser in comparison to the change induced by pH (0.19–0.71 < 0.99). Significant changes in the tertiary structure of α-LA were observed in the presence of 300 mM salts. The Mg^2+^ has a profound disrupting effect on the tertiary structure of α-LA (WSD = 2.13) at *t* = 0 min (Fig. 4), which may explain the abovementioned observations. Contrarily, 300 mM Ca^2+^ stabilized α-LA's tertiary structure (WSD = 0.68), justifying shorter fibrils at the end of the first sigmoidal curve and agreeing with the previously reported data on the stabilizing effect of Ca^2+^ towards α-LA [3, 4].

Because of the complexity of proteins, various interactions between protein and salt may coincide. Therefore, it is problematic to determine which interactions are essential for the particular protein behavior [45]. Despite this fact, we have demonstrated that studied cations significantly influence amyloid aggregation of α-LA, affecting the fibrillization kinetics, changing the protein secondary and tertiary structure, and, in particular cases, the morphology of resulting amyloid aggregates. The effect is more pronounced at higher salt concentrations (300 mM). Regardless of α-LAs preferential binding of Ca^2+^ cations, increasing its initial stability, our results show that the presence of Ca^2+^ is not enough to inhibit its amyloid aggregation.

### Conclusions

We conclude that the properties such as size and hydration of cations coupled with concentration profoundly affect the α-LA's amyloid aggregation propensity and the resulting morphology of the aggregates. While small and strongly hydrated cations (Ca^2+^ and Mg^2+^) significantly accelerate the fibril formation process, larger (excluding Na^+^) and weakly hydrated cations (Na^+^, K^+^, NH_4_^+^, Cs^+^) form fibrils slower; however, resulting in morphologically more uniform longer, individual fibers. These findings are essential to uncover the general mechanism of amyloid fibrillation and the possible future application of such fibers in biotechnology.

## Supplementary Information

Below is the link to the electronic supplementary material.

Supplementary file1 (PDF 864 KB)
