# Supplementary material for: The influence of cations on α-lactalbumin amyloid aggregation
Source: J Biol Inorg Chem. 2022 Sep 23;27(7):679–89. doi: 10.1007/s00775-022-01962-3 (PMC9569292; doi:10.1007/s00775-022-01962-3)
Supplement: Supplementary file 1 — Supplementary file1 (PDF 864 KB) [file 775_2022_1962_MOESM1_ESM.pdf]

The influence of cations on α-lactalbumin amyloid aggregation

Andrea Antosova[1], Miroslav Gancar[1], Zuzana Bednarikova[1], Jozef Marek[1], Eva Bystrenova[2*] and Zuzana Gazova[1*]

[1]Institute of Experimental Physics Slovak Academy of Sciences, Watsonova 47, 040 01 Kosice, Slovakia
[2]Consiglio Nazionale delle Ricerche - Istituto per lo Studio dei Materiali Nanostrutturati (CNR-ISMN), via P. Gobetti 101, 40129 Bologna, Italy.
gazova@saske.sk, eva.bystrenova@cnr.it

Supporting information

Table S1. Schematic representation of studied cation series along with their physicochemical parameters in water. *R* stands for ionic radii in water, and $-\Delta g_{hydr}$ denotes hydration-free energy [17,18].

**Cation**	**Mg^{2+}**	**Ca^{2+}**	**Na^{+}**	**K^{+}**	**NH_4^{+}**	**Cs^{+}**
	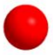	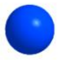	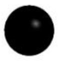	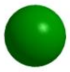	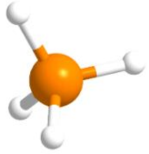	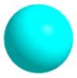
**R [nm]**	0.065	0.099	0.095	0.133	0.148	0.169
**$-\Delta g_{hydr}$ [kJ. mol^{-1}]**	2532	2209	722	651	639	605

Table S2. Kinetic parameters derived from aggregation kinetics of α-LA in the presence of 300 mM salts. Every experiment was performed as a triplicate, and the final data represent an average value accompanied by a corresponding deviation. $t_{Lag}^{1/2}$ represents lag times of aggregation, $t_{Half}^{1/2}$ stands for half-times of aggregation, and $k_{Agg}^{1/2}$ are aggregation rate constants for the first and second sigmoid curves, respectively. * denotes high deviations due to an insufficient dataset in related parts of kinetic curves since the experimental procedure did not allow further data points collection. However, kinetic data mainly served to qualitatively compare different observed processes (single vs. double sigmoidal curves); therefore, we consider them relevant.

**300 mM salts**	**t_{Lag}^{1} [min]**	**t_{Half}^{1} [min]**	**k_{Agg}^{1} [min^{-1}]**	**t_{Lag}^{2} [min]**	**t_{Half}^{2} [min]**	**k_{Agg}^{2} [min^{-1}]**
**$MgCl_2$**	3.7 ± 0.9	9.0 ± 0.4	0.41 ± 0.06	27.8 ± 2.1	29.6 ± 0.9	0.90 ± 1.42*
**$CaCl_2$**	3.6 ± 1.4	8.3 ± 0.7	0.43 ± 0.09	26.7 ± 5.5	38.9 ± 5.3	0.20 ± 0.51*
**NaCl**	5.9 ± 0.6	9.9 ± 0.3	0.46 ± 0.17	26.2 ± 0.9	28.1 ± 0.8	1.32 ± 0.27
**KCl**	5.7 ± 0.4	8.5 ± 0.3	0.71 ± 0.10	18.4 ± 2.8	20.1 ± 0.8	1.11 ± 1.88*
**NH_4Cl**	6.0 ± 0.2	7.8 ± 0.2	1.11 ± 0.15	18.0 ± 0.8	19.4 ± 0.3	1.51 ± 0.57*
**CsCl**	6.1 ± 0.7	8.7 ± 0.3	0.81 ± 0.15	15.6 ± 2.7	20.6 ± 1.3	0.41 ± 0.16

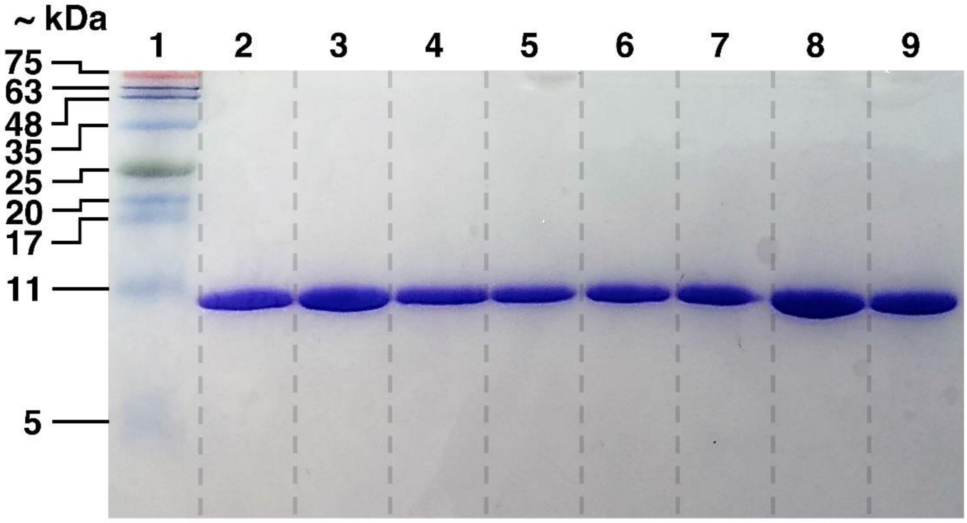

Figure S1. Content of α-LA samples from the first plateau phase separated by SDS-PAGE (12 %). Samples were prepared in 300 mM salts: $MgCl_2$ (**band 4**), $CaCl_2$ (**band 5**), NaCl (**band 6**), KCl (**band 7**), NH_4Cl (**band 8**) and CsCl (**band 9**), pH 2.0 at 65 °C, constant stirring at 1200 rpm for 13-20 min and afterwards solubilized in loading buffer under non-reducing (-βME) conditions. Bands 2 and 3 correspond to the control samples: α-LA, pH 2.0, and pH 5.6 (t = 0 min), respectively. Band 1 denotes the molecular weight marker in kDa.

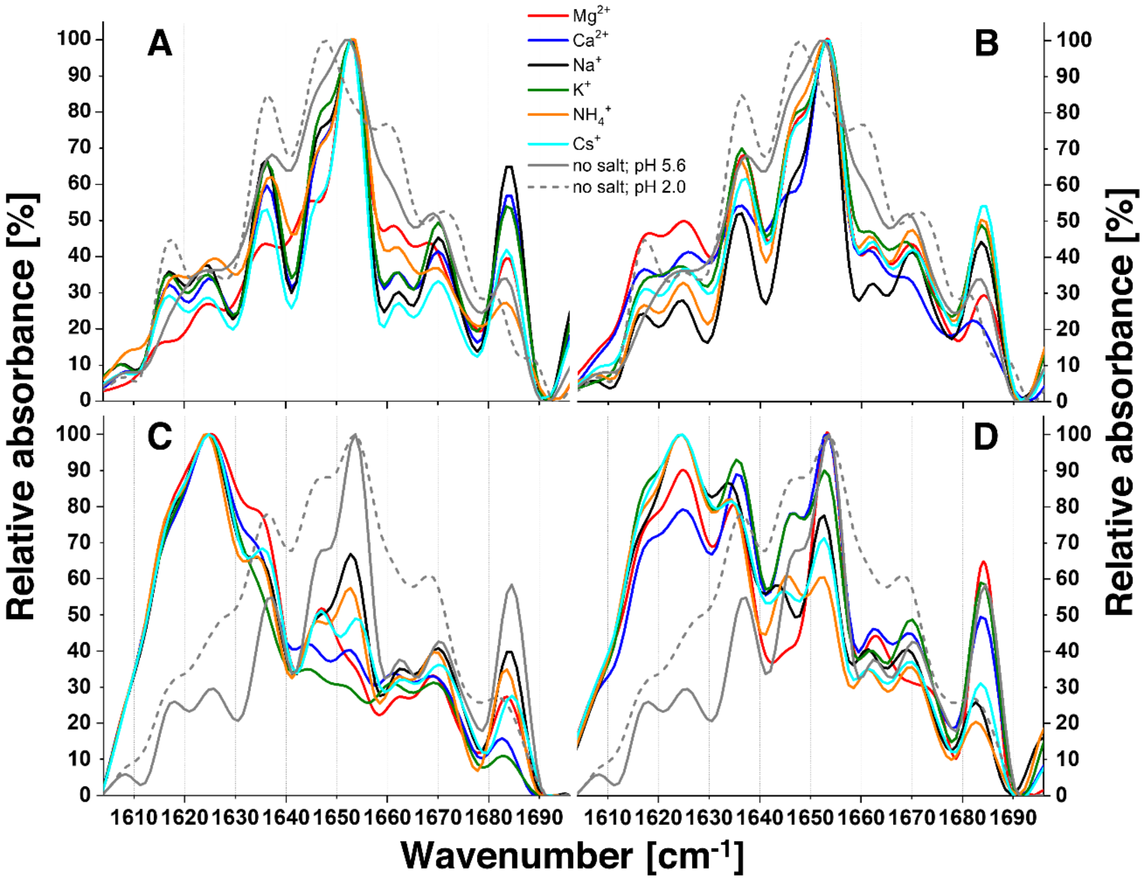

Figure S2. ATR-FTIR spectra of 5 mg.ml^{-1} α-LA in the presence of 100 mM (**A**, **C**) and 300 mM (**B**, **D**) salts were recorded at the start of the fibrillization process (t = 0 min) (**A**, **B**) and after 75 min incubation (**C**, **D**).

Table S3. Secondary structure percentage distributions of α-LA samples in the presence of 100 mM salts measured by the ATR-FTIR at times t = 0 (**A**) and 75 min (**B**), and in the absence of salts at pH 2.0 and 5.6; t = 0 and 75 min (**C**, **D**; taken as control).

100 mM salts	Secondary structure	α-helix	3,10-helix	β-sheet	β-turn	unordered	other
	t [min]	[%]					
**A**							
$MgCl_2$	0	25.3	9.2	27.0	25.7	11.7	1.1
$CaCl_2$		22.5	3.8	29.7	29.1	13.0	1.9
NaCl		20.4	3.4	30.5	29.7	13.7	2.3
KCl		21.4	3.7	29.8	29.4	13.4	2.3
NH_4Cl		22.6	3.2	33.5	24.0	12.7	4.0
CsCl		24.5	3.6	30.5	26.5	1;2.6	2.3
**B**							
$MgCl_2$	75	7.8	1.5	63.5	16.2	9.0	2.0
$CaCl_2$		8.3	4.9	61.3	14.0	9.8	1.7
NaCl		12.5	3.8	55.1	18.3	8.0	2.3
KCl		7.7	5.6	65.2	13.2	6.5	1.8
NH_4Cl		11.2	4.6	57.1	16.1	8.6	2.4
CsCl		9.6	4.1	58.8	15.7	9.5	2.3
**C**							
H_2O; pH 2.0	0	8.2	11.0	30.7	21.3	27.8	1.0
H_2O; pH 5.6		22.2	11.5	33.1	21.5	10.8	0.9
**D**							
H_2O; pH 2.0	75	22.4	2.8	32.8	24.7	16.3	1.0
H_2O; pH 5.6		23.2	6.2	27.8	27.4	14.2	1.2

Table S4. Secondary structure distributions of α-LA samples in the presence of 300 mM salts measured by the ATR-FTIR at times t = 0 (**A**) and 75 min (**B**), and in the absence of salts at pH 2.0 and 5.6; t = 0 and 75 min (**C**, **D**; taken as control).

300 mM salts	Secondary structure	α-helix	3,10-helix	β-sheet	β-turn	unordered	other
	t [min]	[%]					
**A**							
$MgCl_2$	0	20.4	2.9	36.7	23.4	12.9	3.7
$CaCl_2$		22.9	3.8	36.3	21.8	12.0	3.2
NaCl		20.8	5.9	32.1	25.6	14.4	1.2
KCl		25.0	3.5	26.9	30.3	12.9	1.4
NH_4Cl		22.4	3.7	27.7	30.5	13.9	1.8
CsCl		21.5	6.3	30.4	25.9	13.4	2.5
**B**							
$MgCl_2$	75	15.3	9.3	47.2	15.1	7.4	5.7
$CaCl_2$		15.3	2.8	43.7	21.3	12.0	4.9
NaCl		13.6	3.0	47.9	20.2	10.4	4.9
KCl		13.5	3.8	53.9	16.9	8.5	3.4
NH_4Cl		10.8	4.5	54.6	14.7	10.5	4.9
CsCl		12.6	3.2	55.0	16.0	8.2	5.0
**C**							
H_2O; pH 2.0	0	8.2	11.0	30.7	21.3	27.8	1.0
H_2O; pH 5.6		22.2	11.5	33.1	21.5	10.8	0.9
**D**							
H_2O; pH 2.0	75	22.4	2.8	32.8	24.7	16.3	1.0
H_2O; pH 5.6		23.2	6.2	27.8	27.4	14.2	1.2

Table S5. Secondary structure distributions obtained by deconvolution of ATR-FTIR spectra of α-LA samples in the presence of 300 mM salts at times corresponding to the first plateau phase (end of the first sigmoid) of amyloid fibrillization.

300 mM salts	Secondary structure	α-helix	3,10-helix	β-sheet	β-turn	unordered	other
	t [min]	[%]					
$MgCl_2$	20	17.4	3.5	37.4	27.1	11.5	3.1
$CaCl_2$	16	12.1	4.4	46.1	21.9	13.0	2.5
NaCl	16	14.1	5.0	43.0	20.1	11.3	6.5
KCl	13	16.0	4.0	44.3	20.9	10.1	4.7
NH_4Cl	13	16.8	5.7	43.3	22.1	9.6	2.5
CsCl	13	14.9	3.4	50.7	22.1	6.0	2.9

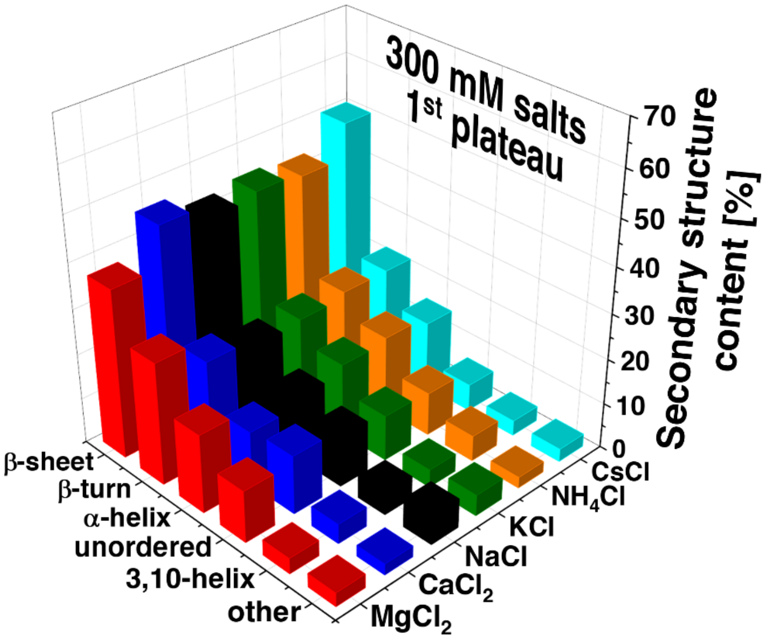

Figure S3. Representation of secondary structure distribution of α-LA samples in the presence of 300 mM salts: $MgCl_2$ (**red bars**), $CaCl_2$ (**blue bars**), NaCl (**black bars**), KCl (**green bars**), NH_4Cl **(orange bars**), and CsCl (**cyan bars**) at times corresponding to the first plateau phase (Table S6).
